# Botryoid Sarcoma of the Vagina

**DOI:** 10.1038/bjc.1961.28

**Published:** 1961-06

**Authors:** R. Salm

## Abstract

**Images:**


### BOTRYOID SARCOMA OF THE VAGINA

R. SALM

*From the Rivers Pathological Department, Camborne-Redruth Hospital, Redruth, Cornwall*

Received for publication February 9, 1961

BOTRYOID sarcoma of the vagina in children is a rare tumour and any one observer may well not encounter a single case during his working life. Thus opinions expressed on the tumour's natural history based on single cases may not reflect the properties of the majority. To this circumstance one must probably attribute statements in even the more recent literature that sarcoma botryoides is a tumour which is mainly locally invasive and rarely metastasizes (Duncan and Fahmy, 1953 ; Ulfelder and Quan, 1947 ; Sharp and Helwig, 1959 ; Taylor, 1958 ; MacGregor, 1960). It would appear likely that this misconception is due to one of two circumstances. In the first place local spread may cause compression of the ureters resulting in hydronephrosis and death due to uraemia before the growth has had time to produce metastatic deposits. Secondly, the diagnosis is made not infrequently in hospital, but the patient is allowed to return home and as often no autopsy can be arranged, no records are made available as to the ultimate features. On the other hand it must be admitted that some botryoid sarcomas do run a protracted course and metastasize late. Thus Daniel, Koss and Brunschwig (1959) recorded a case (their case 5) in which death occurred 6 years after the appearance of the first symptoms.

This report presents a single case of this distressing tumour type, in which full clinical and necropsy data have been obtained, demonstrating widespread dissemination of growth.

### CASE HISTORY

A girl then aged 2 years was taken by her mother to see the family doctor in April 1959, as on occasions some ill-defined structures had been appearing in the introitus vulvae. The physician was unable to detect anything abnormal on external examination and referred the patient to the gynaecologist. He also was unable to find any abnormality on ordinary examination, but repeating his examination under an anaesthetic he noticed several small polypoidal structures high up in the vaginal vault, and these were removed for histological examination. After a diagnosis of botryoid sarcoma had been returned the problem of therapy was considered. After consulting various authorities in this country and abroad it was decided to apply radium intravaginally, and 6000 r were given in 5 days. Ten days after removal of the applicator a large polypoidal structure of irregular shape was expelled from the vagina. Apart from a moderate degree of anaemia with haemoglobin estimations of about 60 per cent the child appeared to be in good health. The parents were fully informed of the position before the patient was discharged and she was nursed, very adequately, at home by her mother. Her abdomen was noted to enlarge gradually, which the family doctor thought to be due to ascites.

Her condition slowly deteriorated and death occurred in November 1959, 7 months after the mother had first become suspicious, and 4 months after the diagnosis had been established in hospital.

*Necropsy*

This was performed 18 hours after death. The abdomen was grossly distended and tense, with protruding umbilicus. A network of distended subcutaneous veins was discernible on the anterior chest wall, which communicated with distended veins running perpendicularly on either side of the midline across the abdomen. The vulval skin was swollen and oedematous, and soft tumour tissue was palpable just behind the introitus.

On opening the abdominal cavity a large tumour mass presented which proved to be neoplastic omentum, measuring 20 × 15 × 4 cm. and weighing 1110 g. There was no ascites, and obstruction of the intra-abdominal venous drainage, as indicated by the ancillary subcutaneous venous plexuses, must have been due to compression of the large abdominal veins by tumour masses from without, as their lumina were patent throughout. After separating some adhesions with the anterior abdominal wall the tumourous omentum could be turned upward and the entire parietal peritoneum was seen to be covered by a smooth, firm, whitish sheet of tumour tissue, measuring 3–7 mm. in width, which had also spread along the mesentery, was enveloping the large gut, liver and spleen, and was encroaching upon the wall of the small gut, especially the ileum and lower jejunum (Fig. 1). The pelvic organs were removed *en bloc* and bisected sagittally. Altogether they weighed 2750 g. The vagina was grossly distended, measuring 6 cm. in diameter, and filled with grape-like tumour masses (Fig. 2). The tumour was found to have spread into the vaginal wall, the cervix, all but the fundal part of the uterine body, the bladder and both parametria, and had given rise to a large tumour mass behind the uterus which had obliterated the pouch of Douglas. The rectal wall was not involved. The distal part of the left tube and the left ovary were identifiable, the right-sided appendages had been incorporated in the retro-uterine mass. In the bladder the growth, which was still covered by intact epithelium, had formed low polypoidal protuberances, producing a cobblestone appearance. The para-aortic lymph glands were grossly enlarged and coalescing, forming tumorous deposits, measuring up to 10 cm. in length. Further involved lymph glands were found in the anterior mediastinum and in the right infraclavicular region. Multiple button-shaped subpleural deposits were present in both lungs. The 4th left rib, near the vertebral column, had been completely destroyed by a single large metastasis, whilst the body of vertebra S I was diffusely invaded by tumour growth. Both renal pelves and ureters were moderately dilated. The ureters were patent to a probe, though some resistance was encountered in their pelvic segments due to compression by tumour tissue from without. Skull and brain, as well as main abdominal organs, were not involved.

*Histology*

*Vaginal biopsy* (59/1555).—The material submitted consisted of a dozen small polypoidal structures, clothed by smooth, glistening epithelium. On microscopical examination they proved to consist of loose, oedematous cellular tissue surrounded by normal stratified epithelium (Fig. 3). The bulky core was made up of small, undifferentiated cells with small hyperchromatic nuclei. Immediately below the epithelium the cells formed a more compact cellular layer. In one of the polypi more cellular areas were discernible, displaying some large, elongated, strap-like cells with strongly eosinophil cytoplasm, and these, with special stains, exhibited well developed cross-striations (Fig. 4). No intercellular mucin was demonstrable.

*Post-mortem material* (59/2680).—Nineteen blocks were cut from various sites. The sections from all showed a cellular pleomorphic sarcoma, the cells of which usually lacked any differentiation. Only after prolonged search a few cells were found in sections from the omental mass, which displayed definite cytoplasmic cross-striation (Fig. 5). Mitotic figures were present throughout in moderate numbers. On the hepatic and splenic surfaces the tumour layer was superimposed loosely onto the intact capsules. In the 4th left rib and in S I the neoplasm had grown diffusely, replacing the bone marrow and resulting in partial or complete destruction of bone trabeculae. The tumour displayed a pronounced tendency to form polypoidal masses on the surface of hollow organs, such as bladder, cervix and vagina, whilst the same tendency was noted in preformed microscopical cavities, such as bronchi (Fig. 6), alveoli and lymph gland sinusoids (Fig. 7). In lymph glands and bone marrow multinucleated tumour giant cells were conspicuous.

### DISCUSSION

The main macroscopical and microscopical features of the present case are those of botryoid sarcoma of the vagina in children. Uncommon findings were the demonstration of polypoidal ingrowths into preformed microscopical cavities such as bronchi, lung alveoli and sinusoids of lymph glands, the presence of multinucleated tumour giant cells in some of the metastatic deposits, the formation of large intra-abdominal metastatic masses, the marked coelomic spread, and the widespread dissemination via lymphatics and bloodstream. As is usually the case the wall of the gut, and especially the rectal wall, were spared, and the ureters, though compressed from without, were not involved.

Grape-like mesenchymal tumours of fairly similar macroscopical and microscopical appearances, including rhabdomyoblasts, and of high malignancy have

---

### EXPLANATION OF PLATES

FIG. 1.—Opened abdominal cavity with tumorous omentum turned upwards. Smooth tumour layer lining parietal peritoneum, covering fundus of bladder and uterus, and encroaching upon serosal aspect of small gut. About $\times \frac{1}{2}$.

FIG. 2.—Bisected pelvic organs showing thickened bladder and vaginal walls and retro-uterine mass ; distended vagina filled with grape-like tumour masses ; and polypoidal appearance of bladder lining. About $\times \frac{1}{2}$.

FIG. 3.—Vaginal biopsy showing polypoid tumour clothed by normal stratified epithelium. Cellular cambium layer underneath, followed by loose-textured oedematous tumour tissue. Haematoxylin and eosin. $\times 23$.

FIG. 4.—Vaginal biopsy, high power field with rhabdomyoblast. Phosphotungstic acid and haematoxylin. $\times 473$.

FIG. 5.—Composite picture of tumour cells from omental mass showing cross-striation. Phosphotungstic acid and haematoxylin. $\times 473$.

FIG. 6.—Lung metastasis showing polypoidal ingrowth into small bronchus. Bronchial epithelium intact. Haematoxylin and eosin. $\times 26$.

FIG. 7.—Invaded abdominal lymph gland with polypoidal ingrowth into peripheral sinusoid. Haematoxylin and eosin. $\times 23$.

1

2

Salm.

3

4

a 5 b

6

7

Salm.

been observed by several investigators as arising at various sites of the head and neck in children and adolescents: in the nasopharynx, soft palate and tonsil; meatus, middle ear and area of Eustachian tube; parotid, orbit and tempero-zygomatic region (Nicory, 1923; Martin and Alexander, 1924; Söderberg, 1933; Cappell and Montgomery, 1937; Maconie, 1944; Stobbe and Dargeon, 1950; St. John and Wood, 1955; Prior and Stoner, 1957; Horn and Enterline, 1958), and recently identical botryoid sarcomas have been observed to originate in the common bile duct before puberty (Horn, Yakovac, Kaye and Koop, 1955; Farinacci, Fairchild, Sulak and Gilpatrick, 1956), whilst the occurrence of this tumour type in bladder and urethra of both sexes, and in the prostate, though rare, has been known for many years (Eibergen, 1952; Hanbury, 1952; Mostofi and Morse, 1952).

But the great majority of botryoid sarcomas do arise in vagina and cervix, and, less frequently, in the uterine body; in the uterus usually after the age of 20. A single case is on record where the tumour originated in the hymen of a girl aged 3 years (Edwards, Sheboygan and Richardson, 1933).

It is often stated that the grape-like neoplasm of the pelvic area arises in tissues and organs derived from the urogenital sinus. But in view of an interesting report of an anal botryoid sarcoma, mentioned briefly by Ober and Edgcomb (1954) and reported more fully by Sharp and Helwig (1959), it would be more accurate to postulate a derivation from tissues of cloacal origin. Amolsch's (1937) case is often quoted wrongly in this connection, as this was not a botryoid sarcoma of the vulva, but a metastasizing myxoid sarcoma, possibly a fibrosarcoma, of the vulval subcutis.

Considering the identical structure of botryoid sarcomas arising in such different sites as the nasopharynx and other areas of the head, and in the common bile duct, as well as in the pelvic organs, it is difficult to understand why many authors have, and are still adhering to the tenet that botryoid sarcomas of the urogenital tract can only be derived from elements of the Müllerian or Wolffian ducts. Willis (1948) has discussed the unlikelihood of such theories.

The histological picture of botryoid sarcoma is uniform and monotonous. It presents as a small-celled undifferentiated mesenchymal neoplasm, the cells of which, at least in the primary growth, are usually separated by much oedematous fluid. According to some observers mucins may be demonstrated in the latter, but in the present case all special mucin stains have been uniformly negative. Below the epithelium of the polypoidal masses the growth tends to be more cellular, forming what has been termed a cambium layer, as it is reminiscent of the cellular zone interposed between the bark and the wood of a tree. Multinucleated tumour giant cells have been occasionally noted in this tumour in adults, but were present in our case and signify probably no more than rapid cell division.

Botryoid sarcomas in children, as already stated, are completely anaplastic tumours except for the occasional differentiation into rhabdomyoblasts which, incidentally, are rarely present in the advanced stages of the disease. This finding raises the question whether the neoplasm should be regarded as a rhabdomyosarcoma, and this term has in fact been used by some authors. However, reasons can be adduced which make it unlikely that botryoid sarcomas represent striped muscle tumours. In the first place most of them occur in organs and tissues where normally no striated muscle is present. Secondly, the cervical and uterine counterpart in adults may also show differentiation into smooth muscle, fat, cartilage and bone. This suggests a retained pluripotential property of the growing mesenchyme, which for unknown reasons is limited in the tumours of children to primitive rhabdomyoblasts.

Aberrant differentiation is well known in both epithelial and mesenchymal growths : squamous metaplasia can be observed in cancers of the gut, breast, stomach, uterus, ovary, gallbladder and other organs, adenocarcinomata may arise in bladder and renal pelvis (Willis, 1958), uterine fibroids may be transformed into "fatty fibroids", consisting of adipose tissue only, and much fatty tissue can arise in the stroma of adenomas of the thyroid (Willis, 1948), of the parathyroid (Ober and Kaiser, 1958), and thymic overgrowths (Shillitoe and Goodyear, 1960). Bone and cartilage may be formed in lipomata and other benign growths (Plaut, Salm and Truscott, 1959), in tumours of the urinary tract (Pang, 1958) and of the soft tissues (Salm, 1959). All these different lesions are due to an identical process : benign and malignant epithelium and mesenchyme may, especially when proliferating, lose its original characteristics, become undifferentiated, and, when subsequently differentiating anew, do so in an unusual and unexpected direction. Thus the finding of rhabdomyoblasts in juvenile botryoid sarcoma, and of other mesenchymal constituents in the adult form, can be readily explained along these lines and there would appear to be no need to invoke theories like those of embryonal rests or displaced tissue of embryonal ducts.

The therapy of botryoid sarcoma has remained disappointing. Almost all workers agree that the neoplasm is radioresistant and thus radium and radiotherapy are of no avail. Early, adequate, and sometimes heroic surgery appears to be the only way to preserve life. Thus Ober, Smith and Rouillard (1958) reported 2 cases of congenital vaginal tumours. In both girls, at the age of about 2 weeks, the uterus was excised together with the entire vagina, and both patients were alive and well 2 years after the operation. Daniel *et al.* (1959) reviewed a series of 13 cases with 2 successes. One, a girl aged 10 months at the time of the operation, was alive and well 2 years later ; the other, a woman of 21 years, had remained well for 5 years. In both patients a total pelvic exenteration had been performed, with bilateral uretero-sigmoidostomy. But it is evident that the prognosis in most cases is unfavourable.

### SUMMARY

A case of vaginal botryoid sarcoma in a girl of 2 years, who died 7 months after the onset of the disease, is presented. A complete necropsy showed much direct and coelomic spread, with massive metastatic tumour formation in omentum and pouch of Douglas, as well as considerable lymphatic and haematogenous dissemination.

In addition to a macroscopical tendency to polypoidal growth there was evidence of polypoidal ingrowths into microscopical preformed cavities, such as lymphatic sinusoids, bronchi and lung alveoli.

The relevant literature is reviewed and the similarity is stressed between botryoid sarcomas of the urogenital region and certain tumours of other sites, especially nasopharynx, middle and external ear and other areas of the head, and of the extrahepatic biliary ducts.

As botryoid sarcomas in the pelvic region arise in the organs of the lower urogenital system, hymen and anus, it must be held that the tumour is formed by tissues derived from the embryonal cloaca, and the occurrence of rhabdomyoblasts and other mesenchymal elements is interpreted as aberrant differentiation.

The therapy is briefly discussed.

I am most grateful to Professor R. A. Willis for reading the manuscript and for his constructive criticisms. I am indebted to Miss Phyllis E. Coleman for the photographic work and to Mr. J. G. H. Ince for the clinical data and for arranging the autopsy.

### REFERENCES

AMOLSCH, A. L.—(1937) *Arch. Path.*, **24**, 777.

CAPPELL, D. F. AND MONTGOMERY, G. L.—(1937) *J. Path. Bact.*, **44**, 517.

DANIEL, W. W., KOSS, L. G. AND BRUNSCHWIG, A.—(1959) *Cancer*, **12**, 74.

DUNCAN, A. S., AND FAHMY, E. C.—(1953) *J. Obstet. Gynaec., Brit. Emp.*, **60**, 87.

EDWARDS, A. C., SHEBOYGAN, W. AND RICHARDSON, A. L.—(1933) *Amer. J. Obstet. Gynec.*, **27**, 896.

EIBERGEN, R.—(1952) *Ned. Tijdschr. Geneesk.*, **96**, 1761.

FARINACCI, C. J., FAIRCHILD, J. P., SULAK, M. H. AND GILPATRICK, C. W.—(1956) *Cancer*, **9**, 408.

HANBURY, W. J.—(1952) *J. Path. Bact.*, **64**, 763.

HORN, R. C. AND ENTERLINE, H. T.—(1958) *Cancer*, **11**, 181.

*Idem*, YAKOVAC, W. C., KAYE, R. AND KOOP, C. E.—(1955) *Ibid.*, **8**, 468.

MACGREGOR, A. R.—(1960) ' Pathology of Infancy and Childhood '. Edinburgh and London (Livingstone), p. 505.

MACONIE, A. C.—(1944) *J. Laryng.*, **59**, 32.

MARTIN, G. E. AND ALEXANDER, W. A.—(1924) *Ibid.*, **34**, 312.

MOSTOFI, F. K. AND MORSE, W. H.—(1952) *J. Urol.*, **67**, 681.

NICORY, C.—(1923) *Brit. J. Surg.*, **11**, 218.

OBER, W. B. AND EDGCOMB, J. H.—(1954) *Cancer*, **7**, 75.

*Idem* AND KAISER, G. A.—(1958) *Ibid.*, **11**, 601.

*Idem*, SMITH, J. A. AND ROUILLARD, F. C.—(1958) *Ibid.*, **11**, 620.

PANG, L. S. C.—(1958) *J. Path. Bact.*, **76**, 357.

PLAUT, G. S., SALM, R. AND TRUSCOTT, D. E.—(1959) *Ibid.*, **78**, 292.

PRIOR, J. T. AND STONER, L. R.—(1957) *Cancer*, **10**, 957.

ST. JOHN, E. G. AND WOOD, Z. P.—(1955) *Radiology*, **65**, 218.

SALM, R.—(1959) *Brit. J. Cancer*, **13**, 614.

SHARP, W. C. AND HELWIG, E. B.—(1959) *Amer. J. Dis. Child.*, **97**, 845.

SHILLITOE, A. J. AND GOODYEAR, J. E.—(1960) *J. Clin. Path.*, **13**, 297.

SÖDERBERG, F.—(1933) *Acta otolaryng., Stockh.*, **18**, 453.

STOBBE, G. D. AND DARGEON, H. W.—(1950) *Cancer*, **3**, 826.

TAYLOR, C. W.—(1958) *J. Obstet. Gynaec., Brit. Emp.*, **65**, 177.

ULFELDER, H. AND QUAN, S. H.—(1947) *Surg. Clin. N. Amer.*, **27**, 1240.

WILLIS, R. A.—(1948) ' Pathology of Tumours '. London (Butterworth), p. 754.—(1958) 'The Borderland of Embryology and Pathology '. London (Butterworth), pp. 516 and 555.


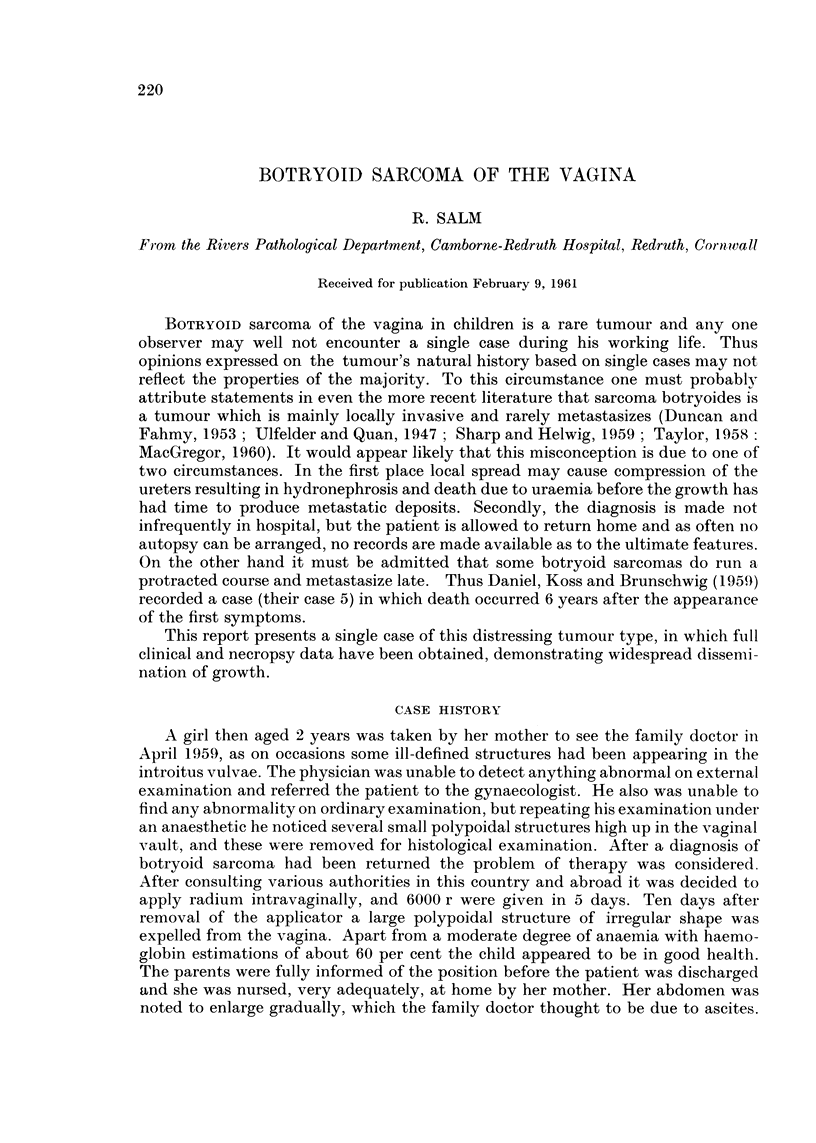

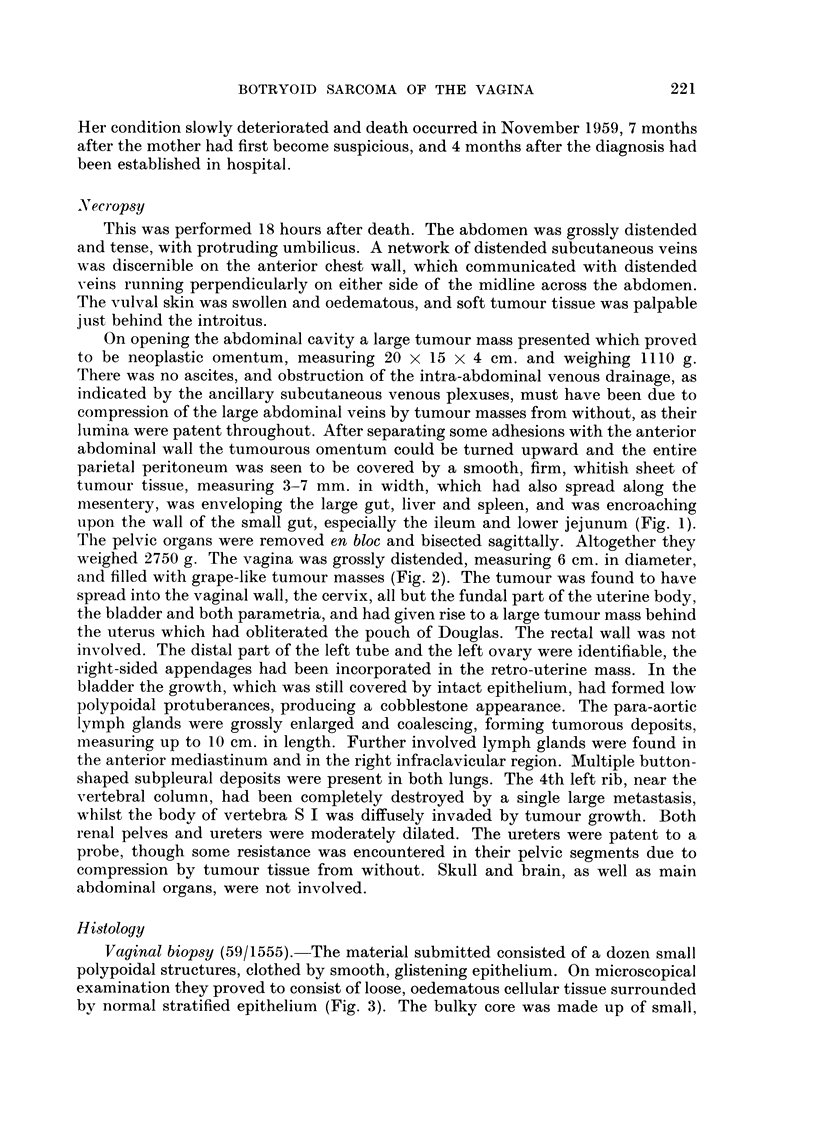

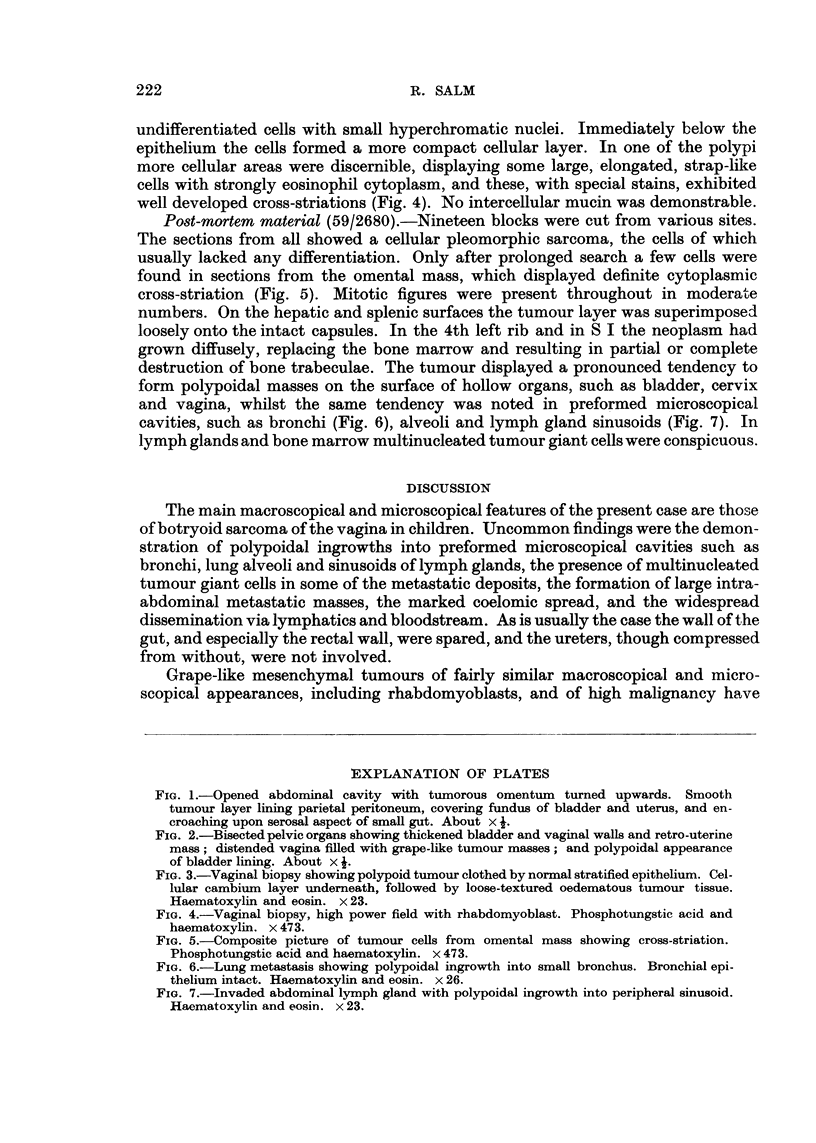

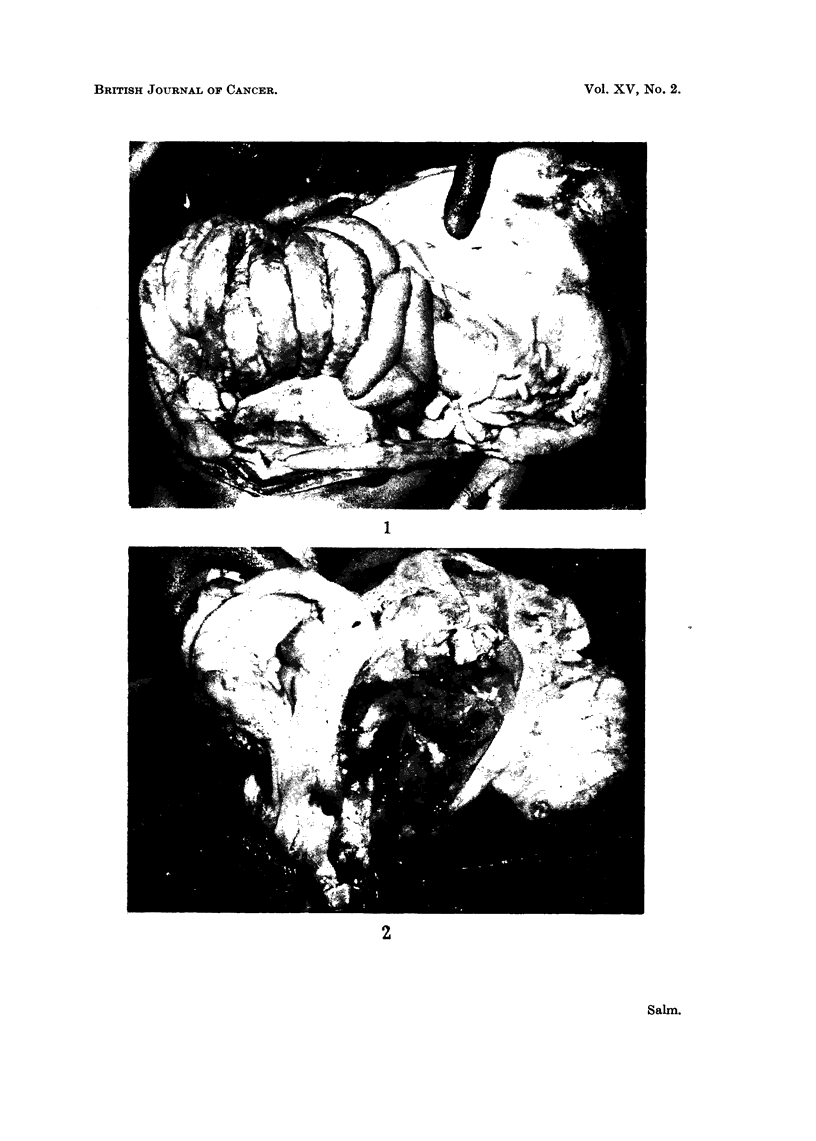

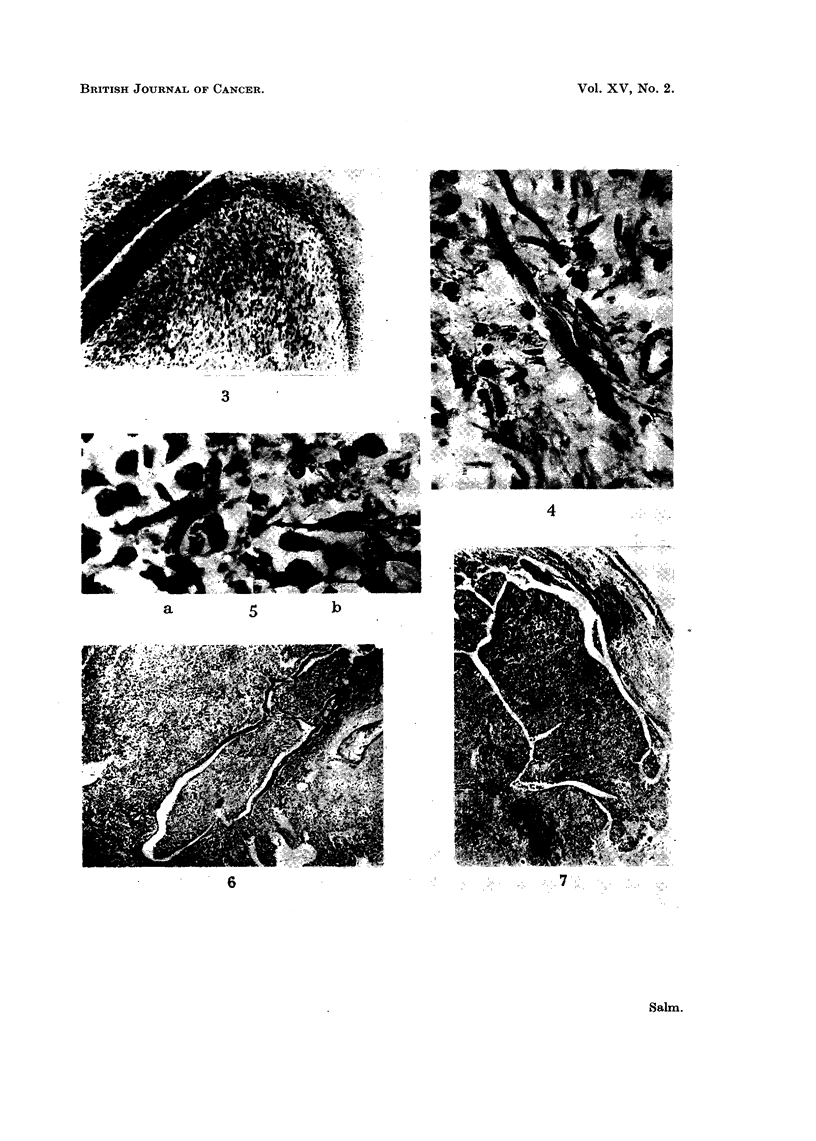

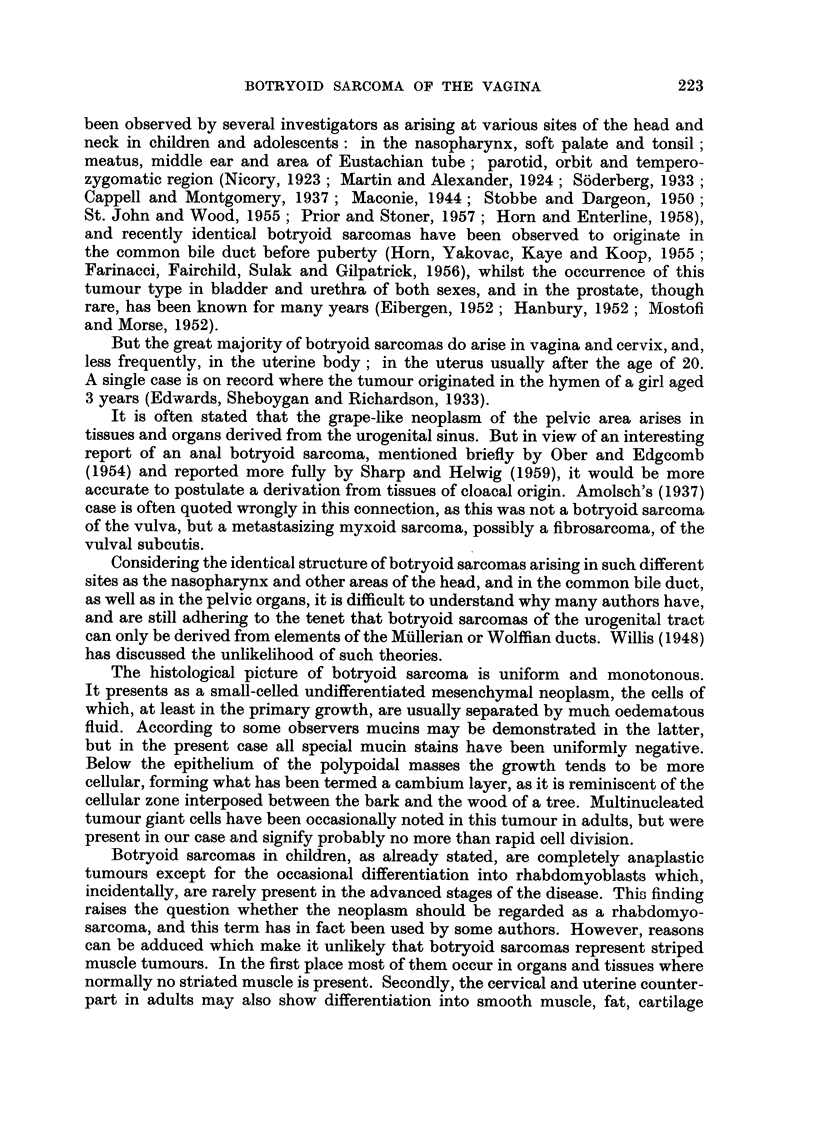

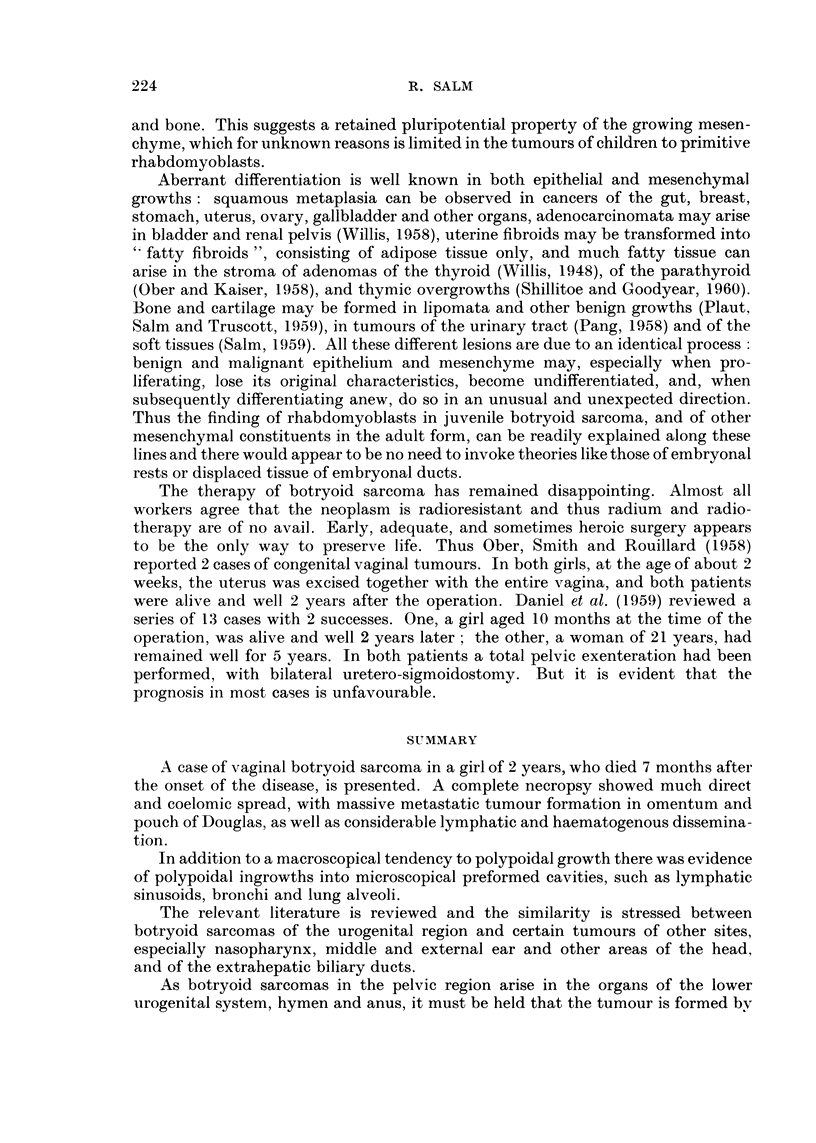

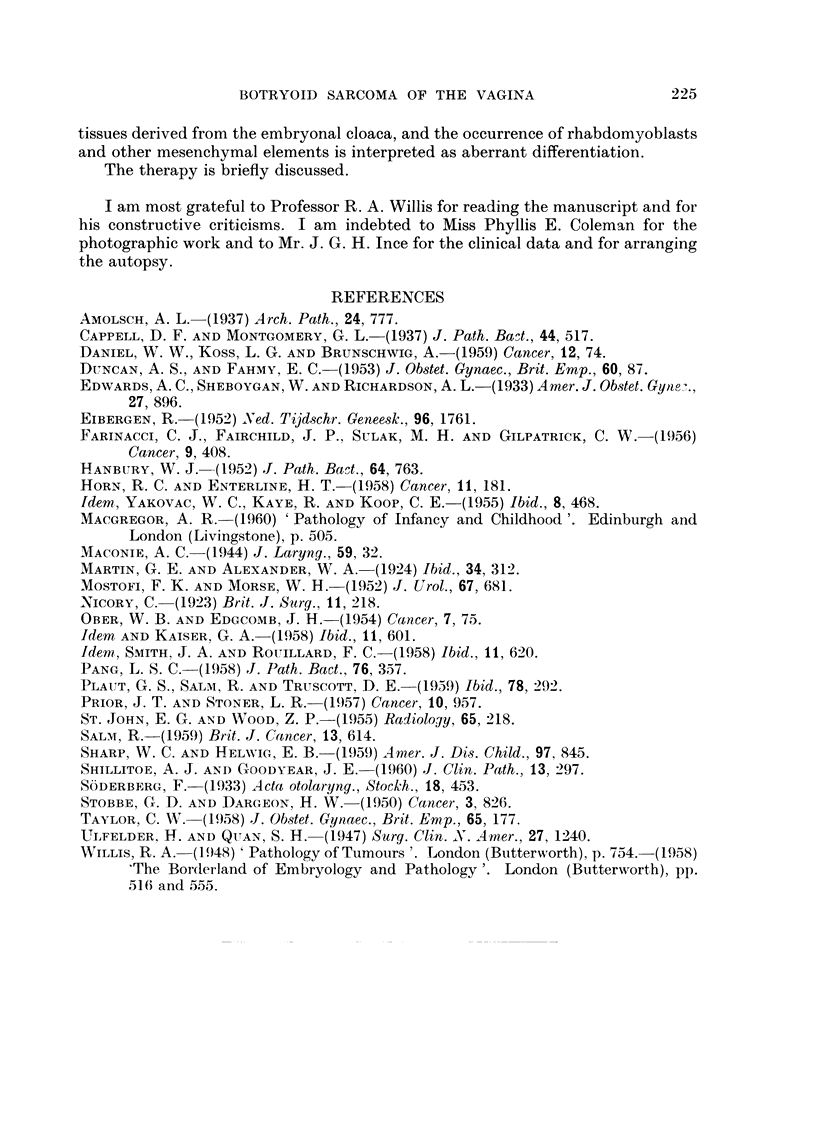


## References

[OCR_00358] EIBERGEN R. (1952). Rhabdomyosarcoma vesicae urinariae.. Ned Tijdschr Geneeskd.

[OCR_00360] FARINACCI C. J., FAIRCHILD J. P., SULAK M. H., GILPATRICK C. W. (1956). Sarcoma botryoides (a form of embryonal rhabdomyosarcoma) of the common bile duct; a report of two cases.. Cancer.

[OCR_00368] HORN R. C., ENTERLINE H. T. (1958). Rhabdomyosarcoma: a clinicopathological study and classification of 39 cases.. Cancer.

[OCR_00378] MOSTOFI F. K., MORSE W. H. (1952). Polypoid rhabdomyosarcoma (sarcoma botryoides) of bladder in children.. J Urol.

[OCR_00380] OBER W. B., KAISER G. A. (1958). Hamartoma of the parathyroid.. Cancer.

[OCR_00392] SALM R. (1959). A case of primary osteogenic sarcoma of extraskeletal soft tissues.. Br J Cancer.

[OCR_00393] SHILLITOE A. J., GOODYEAR J. E. (1960). Thymolipoma: a benign tumour of the thymus gland.. J Clin Pathol.

